# *In Vitro* Activity of the Sudapyridine (WX-081) against Non-Tuberculous Mycobacteria Isolated in Beijing, China

**DOI:** 10.1128/spectrum.01372-22

**Published:** 2022-10-17

**Authors:** Rui Zhu, Yuanyuan Shang, Suting Chen, Hua Xiao, Ruyan Ren, Fen Wang, Yi Xue, Lei Li, Yongguo Li, Naihui Chu, Hairong Huang

**Affiliations:** a National Clinical Laboratory on Tuberculosis, Beijing Key Laboratory on Drug-Resistant Tuberculosis, Beijing Chest Hospital, Capital Medical University, Beijing Tuberculosis and Thoracic Tumor Research Institute, Beijing, China; b Tuberculosis Department, Beijing Chest Hospital, Capital Medical University, Beijing, China; c Shanghai Jiatan Biotech Ltd., a subsidiary of Guangzhou JOYO Pharma Ltd., Shanghai, China; Francis Crick Institute

**Keywords:** Sudapyridine, non-tuberculous mycobacteria, antimicrobial activity

## Abstract

Sudapyridine (WX-081) is a structural analog of bedaquiline (BDQ), which shows an anti-tuberculosis activity but, unlike BDQ, did not prolong QT interval (QT) in animal model studies. This study evaluated the antimicrobial activity of this novel drug against non-tuberculous mycobacteria (NTM). Fifty reference strains of different mycobacterial species, and 132 NTM clinical isolates from four commonly isolated NTM species were recruited. The microplate alamarBlue assay was performed to determine the MIC of WX-081 and BDQ. Cytotoxicity assay was performed for both drugs using the THP-1 cells, and the minimum bactericidal concentrations (MBCs) of both drugs against the reference strains of five selected NTM species were also determined. All the tested reference strains had MICs lower than 0.5 μg/mL, with the majority having MICs far below 0.1 μg/mL for WX-081. The epidemiological cut-offs of WX-081 ranged from 0.0156 μg/mL to 0.25 μg/mL against commonly isolated NTM, and this value was comparable with that of BDQ. The MBC/MIC ratios suggest a bacteriostatic activity for both drugs against the five selected NTM species. Cytotoxicity assays indicated that THP-1 cells had nearly 100% viability when exposed to WX-081 for 24 h below 4 μg/mL, 200- to 300-fold the MICs of Mycobacterium intracellulare, Mycobacterium avium, and Mycobacterium kansasii. WX-081 has a strong antimicrobial activity against different NTM species with low cytotoxicity and therefore has the potential to be used for treating NTM infections.

**IMPORTANCE** Due to the rapidly increased cases globally, non-tuberculous mycobacteria (NTM) disease has become a significant public health problem. Over 200 species or subspecies of NTM have been reported, whereas pulmonary diseases in humans are caused mainly by M. avium complex (MAC), M. kansasii, and M. abscessus. Treatment of NTM infection is often challenging as natural resistance to most antibiotics is quite common among different NTM species. Hence, identifying highly active anti-NTM agents is a priority for potent regimen establishment. The pursuit of new drugs to treat multidrug-resistant-tuberculosis (MDR-TB) may also identify some agents with strong activity against NTM. Sudapyridine (WX-081) is a structural analog of bedaquiline (BDQ), which was developed to retain the antituberculosis efficacy but eliminate the severe side effect of BDQ. This study initially evaluated the antimicrobial activity of this novel drug against non-tuberculous mycobacteria (NTM).

## INTRODUCTION

The rapid increase in global cases has rendered the non-tuberculous mycobacteria (NTM) disease a significant public health problem. In a high-prevalence setting, NTM could account for 30% to 50% of the total isolated mycobacteria (1). Over 200 species or subspecies of NTM have been reported; pulmonary diseases in humans are typically caused by Mycobacterium avium complex (MAC), Mycobacterium kansasii, and Mycobacterium abscessus (2). Treatment of NTM infection is often challenging, as natural resistance to most antibiotics is quite common among different NTM species. Hence, identifying highly active anti-NTM agents is a priority for potent regimen establishment. The pursuit of new drugs to treat multidrug-resistant-tuberculosis (MDR-TB) may also identify some agents with strong activity against NTM (3).

Bedaquiline (BDQ) is a novel antibiotic that targets drug-resistant TB by inhibiting the ATP syntheses (4). In 2019, the World Health Organization (WHO) categorized BDQ as a core drug in the standard treatment regimen of the MDR-TB (5). BDQ demonstrates strong efficacies against MDR TB *in vitro* and *in vivo* and manifests potent activities *in vitro* against many different NTM species (6). Meanwhile, multiple severe side effects of BDQ, such as unexplained mortality and QT prolongation, have been noticed. A meta-analysis of 1,303 patients showed that QT prolongation was 10.6% in clinical use, whereas 0.9% of patients discontinued taking BDQ due to the prolongation of QT (7). The likelihood of side effects, including QT prolongation, hepatotoxicity, and phospholipidosis, limits the use of the BDQ (8). Lu et al. (9) reported that WX-081 had lower cardiotoxicity than BDQ, which resulted in a physiologically appropriate prolongation of the QT interval rather than a significant prolongation. Therefore, modifying the structure of BDQ to discover compounds with comparable efficacy but better safety is becoming essential. Sudapyridine (WX-081) (9) is a new anti-TB drug candidate that originated from the structural optimization of BDQ (10, 11). Previous studies involving animal model-based *in vitro* and *in vivo* assays have shown that WX-081 has comparable efficacy but lower cardiotoxicity and more favorable pharmacokinetic properties than BDQ against TB. Therefore, WX-081 was considered a promising anti-TB drug candidate and is under investigation in a phase 2 clinical trial in China. To better understand the efficacy of WX-081 against NTM, we evaluated its inhibitory activities *in vitro* with clinical isolates of several most-frequently isolated NTM species and analyzed the cytotoxicity. Our study aimed to elucidate the potential of WX-081 for treating NTM infections, especially for the highly prevalent species.

## RESULTS

### MICs of BDQ and WX-081 against NTM reference strains.

The MICs of BDQ and WX-081 against the 50 reference strains are shown in Table 1 and 2. For the same reference strain, the antibacterial activity of WX-081 was similar to BDQ with equal MICs or 1- to 2-fold higher MICs in case of several species (Table 1). Like BDQ, WX-081 showed strong antibacterial activity against almost all the tested slowly growing mycobacterium (SGM) species, with MICs generally much lower than 0.25 μg/mL. Only two SGM species, i.e., *M. rhodesia* and *M. celatum*, had MICs > 2 μg/mL. Furthermore, WX-081 exhibited very potent *in vitro* activity against the recruited RGM reference strains; all of the 26 rapidly growing mycobacterium (RGM) species had MICs ≤0.5 μg/mL, and 23 RGM species had MICs ≤ 0.25 μg/mL.

**TABLE 1:** MICs of BDQ and WX-081 against reference strains of 26 RGM species

Strain by type	Mycobacterial species (strain)	MIC (μg/mL)	
		BDQ^*a*^	WX-081^*b*^
ATCC 19977	Mycobacterium abscessus	0.125	0.25
DSM 45103	Mycobacterium abscessus subsp. Massiliense	0.0625	0.25
ATCC 27406	Mycobacterium agri	0.0156	0.0156
ATCC 27280	Mycobacterium aichiense	0.0625	0.0625
ATCC 23366	Mycobacterium aurum	0.25	0.25
ATCC 33464	Mycobacterium austroafricanum	0.25	0.25
DSM 44177	Mycobacterium brumae	0.0313	0.0625
DSM44017	Mycobacterium confluentis	0.0078	0.0078
ATCC 14472	Mycobacterium chelonae	0.0156	0.125
ATCC 19627	Mycobacterium chitae	0.0625	0.25
ATCC 27278	Mycobacterium chubuense	0.0625	0.125
DSM 44829	Mycobacterium cosmeticum	0.5	0.5
ATCC 19340	Mycobacterium diernhoferi	0.0078	0.0156
ATCC 6841	Mycobacterium fortuitum	0.0078	0.0078
DSM46621	Mycobacterium fortuitum subp. fortuitum	0.0125	0.0125
DSM 44124	Mycobacterium mucogenicum	0.0156	0.0313
DSM44679	Mycobacterium neworleansense	0.0313	0.016
ATCC 27023	Mycobacterium obuense	<0.0078	<0.0078
ATCC 19686	Mycobacterium parafortuitum	0.5	0.5
DSM 43271	Mycobacterium peregrinum	0.0313	0.0625
ATCC 35154	Mycobacterium pulveris	0.5	0.5
ATCC 35796	Mycobacterium senegalense	0.0313	0.0625
ATCC 700731	Mycobacterium septicum	0.03125	0.0625
ATCC 19420	Mycobacterium smegmatis	0.125	0.25
ATCC 19527	Mycobacterium thermoresistibile	0.125	0.25
ATCC 27282	Mycobacterium tokaiense	0.0625	0.0625

a BDQ, bedaquiline.

b WX-081, sudapyridine.

**TABLE 2:** MICs of BDQ and WX-081 against reference strains of 24 SGM species

Strain by type	Mycobacterial species (strain)	MIC (μg/mL)	
		BDQ^*a*^	WX-081^*b*^
ATCC 25276	Mycobacterium asiaticum	<0.0039	<0.0039
ATCC 25291	Mycobacterium avium	0.0078	0.0156
DSM45069	Mycobacterium arosiense	0.0078	0.0156
DSM 44243	Mycobacterium celatum	>2	>2
DSM 44622	Mycobacterium chimaera	<0.0039	<0.0039
ATCC 15754	Mycobacterium gastri	0.125	0.125
ATCC 27726	Mycobacterium gadium	0.0156	0.0156
ATCC 14470	Mycobacterium gordonae	≤0.0039	≤0.0039
ATCC 13950	Mycobacterium intracellulare	0.0078	0.0156
DSM44064	Mycobacterium interjectum	0.0078	0.0078
ATCC 12478	Mycobacterium kansasii	0.0078	0.0156
DSM44627	Mycobacterium kubicae	≤0.0078	≤0.0078
ATCC 927	Mycobacterium marinum	≤0.0039	0.0078
ATCC 19422	Mycobacterium microti	0.0625	0.125
ATCC 19530	Mycobacterium nonchromogenicum	0.25	0.25
DSM 44648	Mycobacterium parascrofulaceum	0.0078	0.0313
ATCC 27024	Mycobacterium rhodesiae	>2	>2
ATCC 19981	Mycobacterium scrofulaceum	0.0156	0.0313
ATCC 35799	Mycobacterium szulgai	0.0126	0.0625
ATCC 33027	Mycobacterium sphagni	0.0156	0.0156
ATCC 27962	Mycobacterium shimoidei	0.25	0.5
ATCC 15755	Mycobacterium terrae	≤0.0039	0.0156
ATCC 23292	Mycobacterium triviale	0.125	0.25
ATCC 19250	Mycobacterium xenopi	0.125	0.25
ATCC 27294	Mycobacterium tuberculosis (H37Rv)	0.0625	0.125

a BDQ, bedaquiline.

b WX-081, sudapyridine.

### MIC distributions and epidemiological cut-offs of BDQ and WX-081 in clinical isolates of different NTM species.

**FIG 1:**
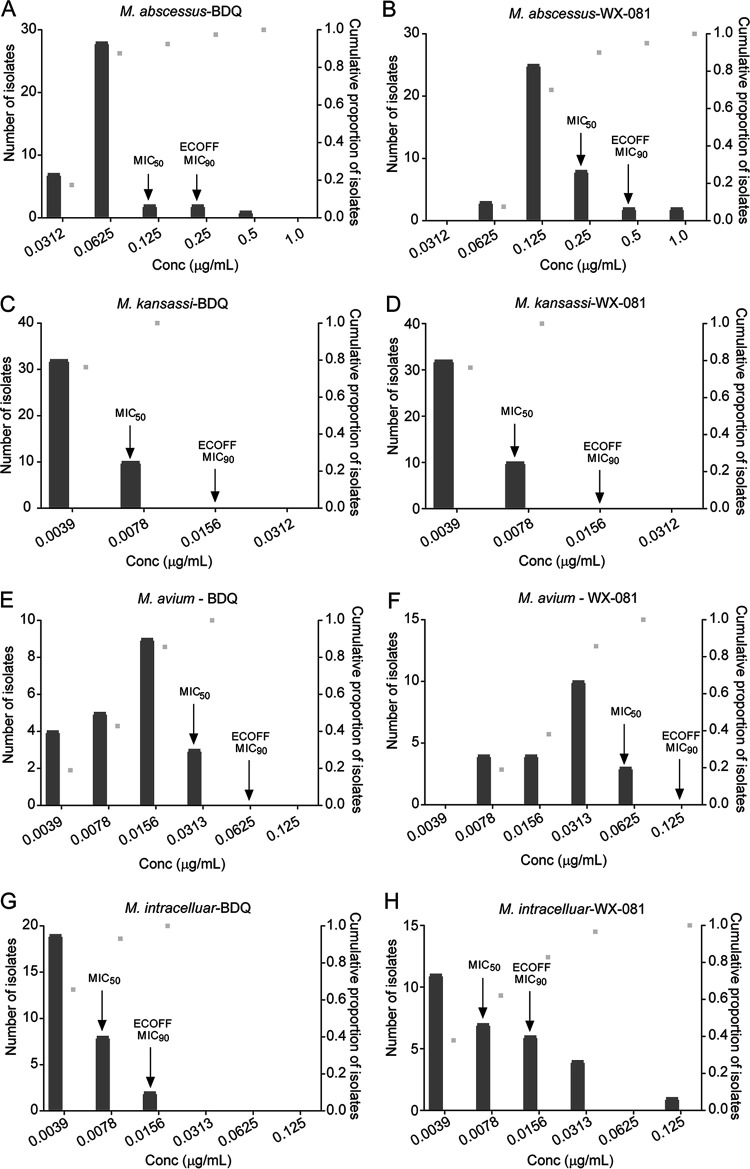
The MIC distributions of BDQ and WX-081 against the four most prevalent NTM species. (A) BDQ MIC distribution for M. abscessus. (B) WX-081 MIC distribution for M. abscessus. (C) BDQ MIC distribution for *M. kansassi*. (D) WX-081 MIC distribution for *M. kansassi*. (E) BDQ MIC distribution for M. avium. (F) WX-081 MIC distribution for M. avium. (G) BDQ MIC distribution for *M. intracelluare*. (H) WX-081 MIC distribution for *M. intracelluare*. Light gray dots represent the cumulative frequency of strains as MIC values change. The bars represent the number of strains corresponding to MIC values.

The MIC distributions of the four most prevalent NTM species to BDQ and WX-081 are shown in Fig. 1. The susceptibility profile of the clinical isolates to BDQ and WX-081 were in concordance with that of the reference strains, i.e., strong antibacterial activity against the absolute majority of the SGM isolates for all the included species. Similar activities were demonstrated against M. abscessus but with relatively higher MIC values than SGM.

BDQ and WX-081 exhibited the strongest activity against M. kansasii and M. intracellulare, with uniform tentative epidemiological cut-off (ECOFF) at 0.0156 μg/mL, and MIC_50_ at 0.0078 μg/mL, and MIC_90_ at 0.0156 μg/mL, respectively. For these two species, the majority of the isolates used in this study had MICs below 0.008 μg/mL. The activity of BDQ and WX-081 against M. avium was weaker than for M. intracellulare, with ECOFF of 0.0625 μg/mL and 0.125 μg/mL, respectively. The inhibitory ability of WX-081 for M. avium was slightly weaker than BDQ. The MIC_50_ and MIC_90_ of BDQ and WX-081 against M. avium were 0.03125 μg/mL and 0.0625 μg/mL, 0.0625 μg/mL and 0.125 μg/mL, respectively. BDQ and WX-081 harbored good activities against M. abscessus with MIC_50_ of 0.125 μg/mL and MIC_50_ of 0.25 μg/mL, respectively. The MIC_90_ of BDQ and WX-081 against M. abscessus was 0.25 μg/mL and 0.5 μg/mL, respectively. The ECOFFs of M. abscessus for BDQ and WX-081 was 0.25 μg/mL or 0.5 μg/mL, respectively.

**FIG 2:**
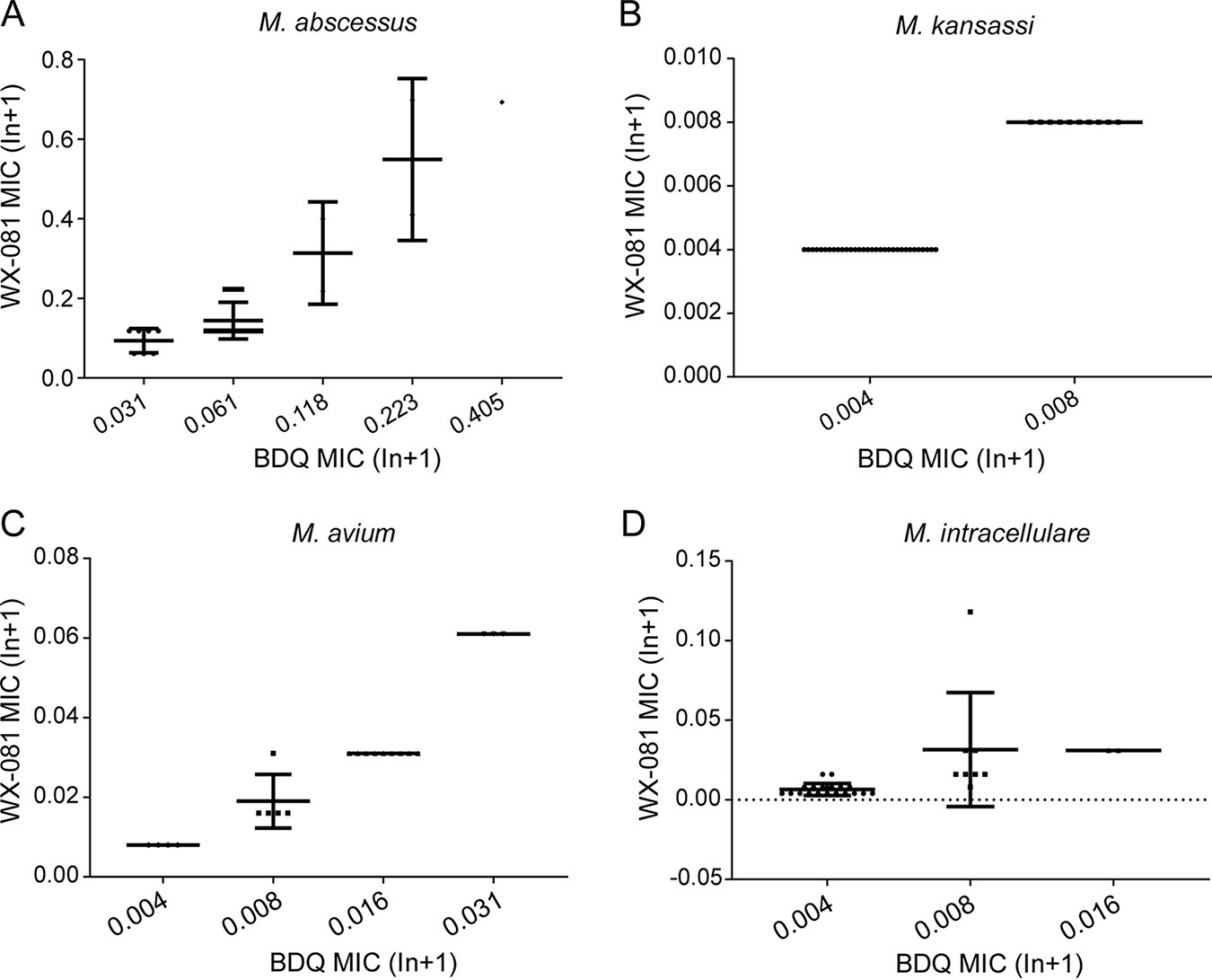
(A) Correlation between MICs of BDQ and WX-081 for M. abscessus. MICs were correlated (*n* = 40, Spearman’s q = 0.722, two-tailed, *P* = 0.0000 < 0.0001. Correlation is significant at the 0.01 level [2-tailed]). (B) Correlation between BDQ MIC and WX-081 MIC for M. kansasii. MICs were correlated (*n* = 42, Spearman’s q = 1.000, two-tailed, *P* = 0.0000 < 0.0001. Correlation is significant at the 0.01 level [2-tailed]). (C) Correlation between BDQ MIC and wx-081 MIC for M. avium. MICs were correlated (*n* = 21, Spearman’s q = 0.967, two-tailed, *P* = 0.0000 < 0.0001. Correlation is significant at the 0.01 level [2-tailed]). (D) Correlation between BDQ MIC and WX-081 MIC for *M. intracellular*. MICs were correlated (*n* = 29, Spearman’s q = 0.784, two-tailed, *P* = 0.0000 < 0.0001. Correlation is significant at the 0.01 level [2-tailed]).

Irrespective of RGM or SGM, the MICs of BDQ and WX-081 had a perfect correlation (Fig. 2). For M. abscessus, BDQ and WX-081 MICs are correlated well (Spearman’s q = 0.722, *P* = 0.0000). The correlation between BDQ MIC and WX-081 MIC for M. kansasii was significant (Spearman’s q = 1.000, *P* = 0.0000). For M. avium and M. intracellulare, the correlation between BDQ MIC and WX-081 MIC was also very strong (Spearman’s q = 0.967, *P* = 0.0000; Spearman’s q = 0.784, two-tailed, *P* = 0.0000).

### Minimum bactericidal concentrations determination.

The minimum bactericidal concentrations (MBCs) of WX-081 and BDQ against the five NTM reference strains were all greater than 32×MIC. Therefore, the MBC/MIC ratios of WX-081 or BDQ against the five reference strains were much higher than 4, suggesting that both drugs are bacteriostatic reagents for M. abscessus, M. fortuitum, M. intracellulare, M. avium, and M. kansasii (Table 3).

**TABLE 3:** The MBC/MIC ratios of SGM and RGM

Category	Species	Agent	MIC (μg/mL)	MBC^*c*^ (μg/mL)	MBC/MIC ratio^*d*^
SGM	*M. kansassi*	BDQ^*a*^	0.0078	0.25	32
		WX-081^*b*^	0.01563	1	64
	M. intracellulare	BDQ	0.0078	0.5	64
		WX-081	0.01563	2	128
	M. avium	BDQ	0.0078	0.5	64
		WX-081	0.01563	1	64
RGM	M. fortuitum	BDQ	0.0078	1	128
		WX-081	0.0078	1	128
	M. abscessus	BDQ	0.125	4	32
		WX-081	0.25	16	64

a BDQ, bedaquiline.

b WX-081, sudapyridine.

c MBC, minimum bactericidal concentration.

d The effect was considered bacteriostatic if the MBC/MIC ratio was higher than 4; otherwise, it was considered bactericidal.

### Cytotoxicity assay of BDQ and WX-081 in the THP-1 cells.

**FIG 3:**
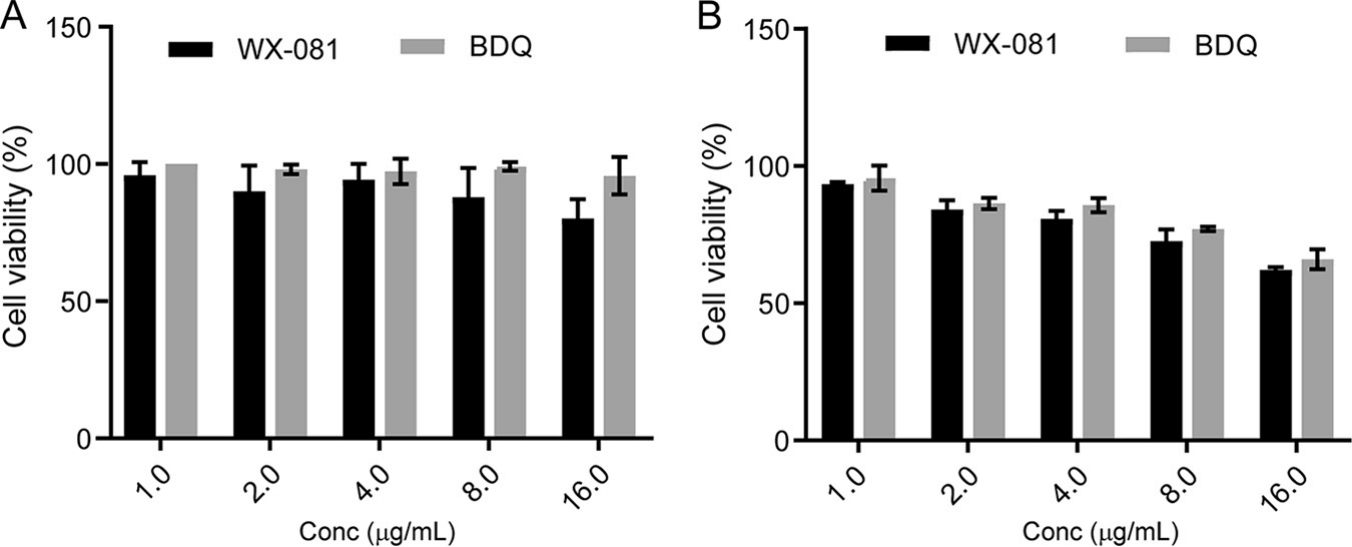
Cytotoxicity assay of BDQ and WX-081 in the THP-1 cells. THP-1 cells were treated with different concentrations of BDQ and WX-081 for 24 h (A) and 48 h (B). The experiments were carried out in quintuplicate. Statistical analysis: two-way ANOVA *P* > 0.05.

When the drug was incubated for 24 h, the viability of THP-1 cells was more than 75% with a concentration of 16 μg/mL, while the viability of THP-1 cells was over 90% at a concentration of 8 μg/mL, and ~100% at a concentration of 4 μg/mL. When incubated with 16 μg/mL drug for 48 h, the cell viability decreased to below 75%, and when incubated with 8 μg/mL drug for 48 h, the cell viability reached over 75%. However, after 48 h of incubation with the drug, the cell viability reached 100% only at 1 μg/mL. Although the cell viability of WX-081 was lower than BDQ, there was no statistical difference between BDQ and WX-081 (Fig. 3).

## DISCUSSION

The potent efficacy of BDQ in MDR-TB treatment has been well recognized. However, the cardiotoxicity of this drug poses a significant concern for its application. A structural analog with comparable efficacy against mycobacteria but better safety would be preferable. WX-081 has recently been reported as a new antibacterial compound with a structure similar to BDQ that, in an animal model, offers less side effects and potential heart liability but retains effectiveness, in contrast to its parental drug. The only publication on this novel drug demonstrated that the MICs of WX-081 against the five susceptible strains ranged from 0.117 to 0.219 μg/mL, and the MICs against 15 drug-resistant strains ranged from 0.026 to 0.966 μg/mL (9). There was better exposure of WX-081 than BDQ in mice and rats, especially in the lung; WX-081 exposure was several times than BDQ at 96 h after dosing. This study first evaluated this novel drug’s anti-mycobacterial activity against NTM strains and demonstrated that both the tested reference strains and clinical isolates were susceptible to it. All the tested reference strains of RGM or SGM species had MICs lower than 0.5 μg/mL, and most of them had MICs far below 0.1 μg/mL (Table 1). Compared with BDQ, these activities were similar, or the MICs were very weakly elevated but remained very low. The inhibitory activity of WX-081 on the clinical isolates of the four most isolated species was in accordance with the outcomes from reference strains. The MIC_50_ and MIC_90_ of M. intracellulare, M. avium, and M. kansasii were ≤0.0625 μg/mL and ≤0.125 μg/mL. These values are very low compared with other known drugs that have been applied in NTM treatment (12). Besides, the MICs of both drugs are closely correlated when analyzed by Spearman’s test (the Spearman’s q ranged from 0.722 to 1). The overall data on reference strains of RGM and SGM and clinical isolates of the most frequently isolated NTM species supported that WX-081 could be as potent as BDQ for the potential application in NTM infection treatment.

ECOFF values for the most prevalent NTM species were defined in this study, which is very important in setting up the breakpoint for drug susceptibility testing in the future. The ECOFFs of M. abscessus, M. intracellulare, M. avium, and M. kansasii are generally very low, ranging from 0.0156 μg/mL to 0.5 μg/mL. These data are consistent with the MIC_50_ and MIC_90_ of each species.

Because WX-081 is an innovative new drug and is yet to be approved for MDR-TB treatment from the Chinese authority of the National Medical Products Administration (NMPA), many profiles of this drug are still underdetermined. Herein, we tested the cytotoxicity of WX-081. In our study, cytotoxicity assays indicated that THP-1 cells had high viability (100%) when exposed to WX-081 ≤ 4 μg/mL. This drug concentration was 16 times higher than the MIC of the reference strain of M. abscessus and 200 to 300 times higher than the MIC of the reference strains of M. abscessus, M. intracellulare, M. avium, and M. kansasii. This favors the possibility of WX-081 as a safe antimicrobial agent against commonly isolated NTM strains. The MBC/MIC ratios of WX-081 or BDQ suggested both drugs have bacteriostatic activity toward M. abscessus, M. fortuitum, M. intracellulare, M. avium, and M. kansasii. In this study, we applied the standard of MBC/MIC ratio ≤4 to determine the bacteriostatic or bactericidal activities. The first publication on WX-081 used a standard of MBC/MIC ratio < 32. They acquired 16 for both WX-081 and BDQ and concluded that both drugs are bactericidal antibiotics for tuberculosis. However, even though we applied this higher value standard, both drugs are still defined as bacteriostatic, highlighting the inherently resistant characteristic of NTM in general.

There are some limitations to our study. First, all the tested clinical isolates were collected from a single institution. It may not represent all the characteristics of the species. Second, the bactericidal activities of WX-081 against different NTM species were only investigated *in vitro*; thus, further *in vivo* evaluation could help to understand the real value of this drug in treating NTM infections.

In conclusion, both the tests on reference strains and clinically isolated NTM demonstrated that WX-081 has a strong antimicrobial activity against different NTM species with low cytotoxicity and, therefore, could be considered a promising drug candidate for NTM infection treatment.

## MATERIALS AND METHODS

### Ethics statement.

As the study only concerned laboratory testing of mycobacteria without the direct involvement of human subjects, ethics approval was not sought.

### Reference strains and clinical isolates.

Fifty mycobacterial reference strains stored in the Bio-bank in Beijing Chest Hospital (Beijing, China) were tested, including 26 RGM and 24 SGM species. These reference strains were obtained either from the American Type Culture Collection (ATCC) or the German Collection of Microorganisms (DSM). The species’ composition of these reference strains is listed in Table 1 and 2. A total of 132 NTM clinical isolates were used, including 40 M. abscessus, 29 M. intracellulare, 21 M. avium, and 42 M. kansasii. The isolates were differentiated into species groups by both growth tests on a *p*-nitrobenzoic acid-containing medium and by sequence alignments of *16S rRNA*, *hsp65*, *rpoB*, *16-23S rRNA* internal transcribed spacer as mentioned previously (13).

### MIC testing.

BDQ were purchased from Liye-Pharmaceutical (Nanjing, China) and WX-081 provided by Shanghai Jiatan Biotech, Ltd. (Shanghai, China). Both drugs were dissolved in dimethyl sulfoxide (DMSO). Stock solutions were aseptically prepared at concentrations of 8 mg/mL. The inoculum was prepared with fresh culture grown on a Lowenstein-Jensen medium. The broth microdilution method was performed according to the guidelines of the Clinical and Laboratory Standards Institute (CLSI) (12). Cation-adjusted Mueller-Hinton broth (CAMHB), enriched with 5% oleicacid–albumin–dextrose–catalase (OADC), was used for SGM, while CAMHB without OADC was used for RGM. The broth microdilution format was set up as a 2-fold dilution, and the concentration ranges were 0.0039to 2.0 mg/L for BDQ and WX-081. Briefly, a bacterial inoculum with a turbidity equivalent to a 0.5 McFarland standard dilution 1:200 was prepared for each strain. Plates were then incubated at 37°C for 7 to 10 days for SGM and 3 days for RGM, respectively. Seventy microliter solution containing 20 μL alamarBlue and 50 μL 5% Tween 80 was added to each well and incubated for 24 h at 37°C before assessing color development. A blue to pink or purple change indicated bacterial growth (14). The MIC value was determined as the lowest drug concentration of antibiotic that prevented a color change from blue to pink.

### Tentative ECOFF determination.

For species with enough isolates and intense inhibitory activity demonstrated by BDQ/WX-081, the ECOFF was determined according to the distribution profile of the MIC values. The ECOFF was defined as the concentration that could inhibit >95% of the bacterial population for the unimodal MIC distribution profile. In contrast, for the bimodal MIC distribution profile, ECOFF was set between the two populations.

### MBCs determination.

MBC against the five reference strains of NTM in 96-well plates was determined by CFU-based enumeration after 3 or 7 days of incubation with relevant compounds. According to the CLSI guidelines (15), MBC was determined by 99.9% kill of the final inoculum. Initial CFU was calculated on the same day when the culture was incubated in 96-well microplates. The drug concentration ranged from 1×MIC to 128×MIC for WX-081 and BDQ. RGM was incubated for 3 days, and SGM was incubated for 7 to 10 days. Culture aliquots of 100 μL of each well were placed on CAMHB with or without 5% OADC for cultivation. CFU was counted after 7 to 10 days for RGM or 28 days for SGM. According to the CLSI guidelines, MBC was defined as the lowest effective drug concentration in the CFU, at least 3log10 lower than the initial CFU. An antibiotic was considered bactericidal when the MBC/MIC ratio was ≤4; otherwise, it was bacteriostatic.

### Cytotoxicity assay.

The toxicity of BDQ and WX-081 was assessed using THP-1 cells. Cells were seeded into 96-well plates and differentiated into macrophages with 100 nM PMA. After 48 h, cells were washed once and cultured in fresh RPMI medium (Gibco) with 10% fetal bovine serum (RPMI complete medium). The solution was added into the wells with final concentrations ranging from 1 to 16 μg/mL and incubated for an additional 24 h and 48 h. The cytotoxicity of different concentrations of BDQ and WX-081 was monitored using a CCK-8 Cell Proliferation and Cytotoxicity assay kit (Solarbio, Beijing, China). The cell culture supernatant was analyzed, and the absorbance value (Abs) under 450 nm was recorded using a Multiskan Go microplate reader (Thermo Fisher Scientific, USA). The cell survival rate (%) at each concentration was determined as follows: cell survival rate = ([Abs450 of treated cells/Abs450 of control cells]/[Abs450 of no treated cells/Abs450 of control cells]) × 100%.

### Statistical analysis.

Data were analyzed using SPSS 23.0 software and GraphPad Prism 7.0 software. Spearman’s test analyzed the correlation between BDQ MIC value and WX-081 MIC value for clinical isolates. A two-way analysis of variance (ANOVA), followed by a *post hoc* test, was used for cytotoxicity assay to determine significant differences between the groups. Differences were considered to be statistically significant for *P* value of <0.05.
